# Takayasu arteritis, an atypical presentation as chronic kidney disease

**DOI:** 10.4314/ahs.v23i1.82

**Published:** 2023-03

**Authors:** Vijayan Neeraja, Vihari Nakka, Meena Mahadev, Midha Naresh, Yadav Taruna, Sakthivadivel Varatharajan

**Affiliations:** 1 All India Institute of Medical Sciences Jodhpur, Department of General Medicine; 2 All India Institute of Medical Sciences Jodhpur, Department of Diagnostic and Interventional Radiology; 3 ALL India institute of Medical Sciences Bibinagar, Department of General Medicine

**Keywords:** Takayasu arteritis, chronic kidney disease, thrombocytopenia

## Abstract

Takayasu arteritis, also known as “pulseless disease” usually affects major vessels like aorta and its branches, pulmonary arteries, and renal arteries. Hypertension is the common presentation. Chronic kidney disease involvement is less common. Only a few chronic kidney disease cases are reported so far in Takayasu arteritis. We discuss a case of a young female who presented with accelerated hypertension with chronic kidney disease with preserved peripheral pulses. The diagnosis was confirmed by Computed tomography aortic angiogram, which showed diffuse circumferential thickening, multifocal ectasia, and aneurysmal dilatation with few saccular outpouchings of the aortic arch, descending thoracic and abdominal aorta (Type V). The patient was treated with steroids, antihypertensives, antiplatelet, and hemodialysis.

## Introduction

Takayasu arteritis is a systemic vasculitis involving major vessels, common in Asian population; with predilection for young female. Its etiology is unknown, but genetic factors and role of infection has been postulated. It has varied presentation, from asymptomatic, absent pulses, abnormal bruit to catastrophic presentation like stroke, malignant hypertension, cardiac failure, and renal involvement. Hypertension is seen in 33-83% of patients, commonly due to renal artery involvement.^1^ Renal involvement is seen 4.5-33% of Indian population.^2^,^3^,^4^

Here we report a case of young female presented with accelerated hypertension with chronic kidney disease (CKD)

## Case report

Twenty-seven-year-old female, a known case of hypertensive for 5 years and CKD for 3 years - not on dialysis, presented to emergency with complaints of blurring of vision, headache and vomiting. On examination, blood pressure was 200/110 mm Hg. All peripheral pulses were equally palpable without radio-femoral or radio-radial delay. Cardiac examination showed a systolic murmur of grade 3 in mitral area. Abdomen examination showed bruit over the epigastric region. Other system examinations were normal. Fundus examination showed grade 4 hypertensive retinopathy without papilledema. Electrocardiogram (ECG) was suggestive of left ventricular hypertrophy. Hemogram revealed anemia (Hemoglobin 8.3gm/dl), thrombocytopenia (platelet count 74000 cells/mm^3^), and normal total leucocyte count (5050 cells/mm^3^). Peripheral blood film showed normocytic normochromic anemia, target cells, tear drop cells, and thrombocytopenia. She had raised erythrocyte sedimentation rate (56 mm/hr), highly sensitive C reactive protein (>6 mg/L) and lactate dehydrogenase (555 U/L). Biochemical analysis revealed abnormal kidney function tests (urea 138 mg/dl, creatinine 6.39 mg/dl, estimated glomerular filtration rate (eGFR 8.8ml/min/1.73m^2^), hyperkalemia (6 mEq/L). Urine analysis showed mild proteinuria (24 hours urine protein 1.36 grams) with hematuria. Patient was diagnosed as a case of accelerated hypertension, CKD- stage V with bicytopenia. Patient was treated with antihypertensive (nifedipine 10 mg three times a day (TDS), clonidine 0.1 mg three times a day (TDS), hemodialysis, and supportive measures.

**Figure 1:**
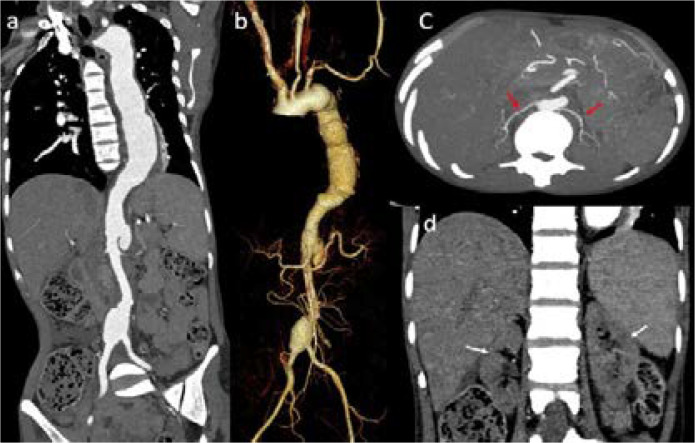
Computed tomography aortic angiogram **Legend:** (a) Coronal image shows diffuse circumferential mural thickening of descending thoracic and abdominal aorta. (b) 3D-Volume rendered (VR) image depicts multifocal ectasia and aneurysmal dilatation of descending aorta with few saccular outpouchings. (c) Axial maximum intensity projection (MIP) image shows bilateral thin calibre renal arteries (red arrows). (d) Coronal image showing bilateral small kidneys (white arrows).

Abdominal ultrasonography with arterial Doppler showed bilateral contracted kidneys with multiple ectatic segments and a fusiform aneurysm (3cm in maximal diameter) proximal to the aortic bifurcation. Computed tomography aortic angiogram showed diffuse circumferential thickening, multifocal ectasia and aneurysmal dilatation with few saccular outpouchings of aortic arch, descending thoracic and adominal aorta, suggestive of Takayasu's arteritis (Figure 1).

Echocardiogram showed left ventricular hypertrophy. Serology for hepatitis B, hepatitis C, and human immunodeficiency virus were negative. Antinuclear antibody (ANA), antineutophil cytoplamic antibodies (p-ANCA, c-ANCA) were negative. Mantoux test was also negative. Prednisolone 1mg/kg/day and low dose aspirin (75mg/day) were added. Patient general condition, renal function, and platelets were improved. Patient was discharged with steroid, aspirin, antihypertensives and maintenance hemodialysis.

## Discussion

Takayasu arteritis, also known as the “pulseless disease” is a systemic large vessel vasculitis, commonly affecting aorta and its branches. The disease commonly presents in the 2^nd^ or 3^rd^ decade of life and may initially display nonspecific inflammatory features like fever, malaise and arthralgia. This will be followed by a chronic phase with development of vascular insufficiency, thrombosis or aneurysms.

American college of rheumatology recommended six criteria for the diagnosis of Takayasu arteritis.^5^ Our patient met the three criteria's, young onset, abdominal bruit and narrowing of bilateral renal arteries, inferior mesenteric artery, and short segment of left common carotid artery. There was no claudication, and carotidynia in our patient. The characteristic features of absent pulses and blood pressure discrepancy were absent in our patient, which are present in 70-80% of patients.^2^

Takayasu arteritis classified into five types depends on the angiographic pattern. (Table 1)^6^

**Table 1:** Angiographic classification of Takayasu arteritis

Type I	Aortic arch and its branches
Type IIa	Ascending aorta, aortic arch and its branches
Type IIb	Ascending aorta, aortic arch and its branches, thoracic descending aorta
Type III	Thoracic descending aorta, abdominal aorta, and/or renal arteries
Type IV	Abdominal aorta and/or renal arteries,
Type V	Combined features of types IIb and IV

Our patient had Type V, involvement of aortic arch, descending, abdominal aorta and renal arteries which is the commonest type in Indian population as per recent study.^7^ Hypertension is the common manifestation of takayasu arteritis in Indian population which may be due to renal artery stenosis, diminished baroreceptor reactivity, and arterial stiffness.^3^ Renal artery injury in the form of stenosis, dilatation and aneurysm is seen in 30-35% of Asian population.^8^ Prolonged ischemia due to renal artery involvement releases inflammatory markers leading to fibrosis, glomerular hyalinization and progressive kidney atrophy.^9^ Our patient presented with young onset hypertension with bilateral contracted kidney, due to renal artery involvement.

Aneurysm and dilatation are seen in 25% of takayasu arteritis, commonly affects aorta, subclavian, renal, carotid, and vertebral arteries.^10^ Our patient had multiple ectatic lesions from aortic arch to renal arteries along with fusiform aneurysm proximal to aortic bifurcation.

Thrombocytopenia in Takayasu arteritis may be due to vasculitis, renal dysfunction, and associated Immune Thrombocytopenia (ITP). Katharine Rainer et al. reported a case of Takayasu arteritis with CKD, and thrombocytopenia as part of renal dysfunction.^11^ Our patient's platelet count improved after steroid therapy suggesting the possibility of coexisting ITP and/- or part of vasculitis.

Medical Management of Takayasu arteritis includes steroids, methotrexate, azathioprine, mycophenolatemofetil, leflunomide, cyclophophamide, Tumor necrosis factor (TNF) alpha inhibitors, Tocilizumab and abatacept. Steroids are the initial treatment of choice.^12^,^13^ Surgical management depends on the arterial territory involvement, includes percutaneous transluminal angioplasty, stent graft placement, and aortic valve replacement.^14^ Thromboprophylaxis with low dose aspirin is essential in all patients.^15^ Our patient was started on oral steroid and low dose aspirin to prevent further cardiovascular mortality.

We described an atypical presentation of Takayasu arteritis in young female with diffuse ectatic involvement of aortic arch to renal arteries with fusiform aneurysm (Type V) with CKD-stage V requiring hemodialysis with preserved peripheral pulses. Early diagnosis is crucial for the initiation of disease modifying therapies and surgical intervention to prevent end organ damage and lifelong morbidity.

## Conclusion

Takayasu arteritis is a rare condition with varied presentation. Early recognition and management will prevent the morbidity and mortality. Takayasu arteritis should be kept in mind when young patient presents with hypertension and CKD.
